# Stabilization of Murine Norovirus by Bacteria

**DOI:** 10.1128/msphere.00046-22

**Published:** 2022-05-09

**Authors:** Melissa R. Budicini, Julie K. Pfeiffer

**Affiliations:** a Department of Microbiology, University of Texas Southwestern Medical Center, Dallas, Texas, USA; University of Kentucky College of Medicine

**Keywords:** murine norovirus, stability, bacteria

## Abstract

Enteric viruses encounter various bacteria in the host, which can impact infection outcomes. The interactions between noroviruses and enteric bacteria are not well understood. Previous work determined that murine norovirus (MNV), a model norovirus, had decreased replication in antibiotic-treated mice compared with conventional mice. Although this suggests that the microbiota promotes MNV infection, the mechanisms are not completely understood. Additionally, prior work with other enteric viruses, such as poliovirus and coxsackievirus B3, demonstrated that virions bind bacteria, and exposure to bacteria stabilizes viral particles and limits premature RNA release. Therefore, we examined interactions between MNV and specific bacteria and the consequences of these interactions. We found that the majority of Gram-positive bacteria tested stabilized MNV, while Gram-negative bacteria did not stabilize MNV. Both Gram-positive and Gram-negative bacteria bound to MNV. However, bacterial binding alone was not sufficient for virion stabilization, since Gram-negative bacteria bound MNV but did not stabilize virions. Additionally, we found that bacteria conditioned medium also stabilized MNV and this stabilization may be due to a small heat-stable molecule. Overall, this work identifies specific bacteria and bacterial components that stabilize MNV and may impact virion stability in the environment.

**IMPORTANCE** Enteric viruses are exposed to a wide variety of bacteria in the intestine, but the effects of bacteria on viral particles are incompletely understood. We found that murine norovirus (MNV) virion stability is enhanced in the presence of several Gram-positive bacterial strains. Virion-stabilizing activity was also present in bacterial culture medium, and activity was retained upon heat or protease treatment. These results suggest that certain bacteria and bacterial products may promote MNV stability in the environment, which could influence viral transmission.

## INTRODUCTION

Noroviruses are a leading cause of nonbacterial gastroenteritis disease around the world. Human norovirus (HuNoV) causes 1 billion infections and 200,000 deaths annually (1–3). The economic impacts of these infections are significant, with $4.2 billion in direct health system costs and $60.3 billion in societal costs globally each year (1, 4). Despite its impact, little is known about the mechanisms of disease of HuNoV due to the lack of a robust cell culture system or small animal model. However, in 2003, a genetically related virus, murine norovirus (MNV), was discovered and is now used as a model system for HuNoV due to its genetic similarity, efficient replication *in vitro*, and tractable mouse models (5, 6). MNV is a small nonenveloped, positive-sense RNA virus with a 7.5-kb genome (7). The main site of MNV infection is the intestine, and it is spread through the fecal-oral route.

During infection in the intestine, enteric viruses encounter 10^14^ bacteria (8). Enteric viruses such as poliovirus, coxsackievirus, and reovirus can interact directly with the gut microbiota to enhance infection through a variety of mechanisms such as increased host cell binding, increased receptor binding, and increased viral stability in the presence of bacteria (9–15). For MNV, depletion of the microbiota by antibiotic treatment in mice decreases viral titers during acute and persistent infection (16, 17). Certain host genes involved in innate immune responses, including those encoding the interferon lambda receptor, Stat1, and Irf3, were required for antibiotic-mediated loss of viral persistence in mice, suggesting that the microbiota promotes MNV persistence by inhibiting host innate responses (17). However, effects of the microbiota on MNV are complex and site specific, since the microbiota can inhibit MNV infection of the upper intestine via bile acid priming of interferon lambda responses (18). MNV can bind directly to bacteria *in vitro* (19, 20). Although these findings suggest that the presence of bacteria is important for promoting MNV infection, the mechanisms are incompletely understood.

Because MNV is spread through the fecal-oral route, virion stability in the environment is necessary for maintenance of viral infectivity and transmission to a new host. Viral stability can be measured by exposing viral particles to high temperatures and quantifying remaining viable viruses. Heat causes changes in viral capsid conformations that can lead to viral genome release and virion inactivation (21). For poliovirus, another enteric virus spread through the fecal oral route, the presence of bacteria and bacterial components such as lipopolysaccharide (LPS) can stabilize the virus capsid, which leads to increased transmission (10). In this study, we determined the effect of bacteria and bacterial components on MNV thermostability. We used heat inactivation assays with whole bacteria, bacterial surface molecules, and conditioned medium and determined their impact on viral stability. We found that specific Gram-positive bacteria and conditioned medium from Gram-positive bacteria stabilized MNV. Conversely, the Gram-negative bacteria tested had little impact on viral stability. Overall, these findings define interactions between MNV and specific bacteria which may provide insight into virion environmental stability and transmission.

## RESULTS

### Most Gram-positive bacteria tested enhance stability of MNV.

**FIG 1:**
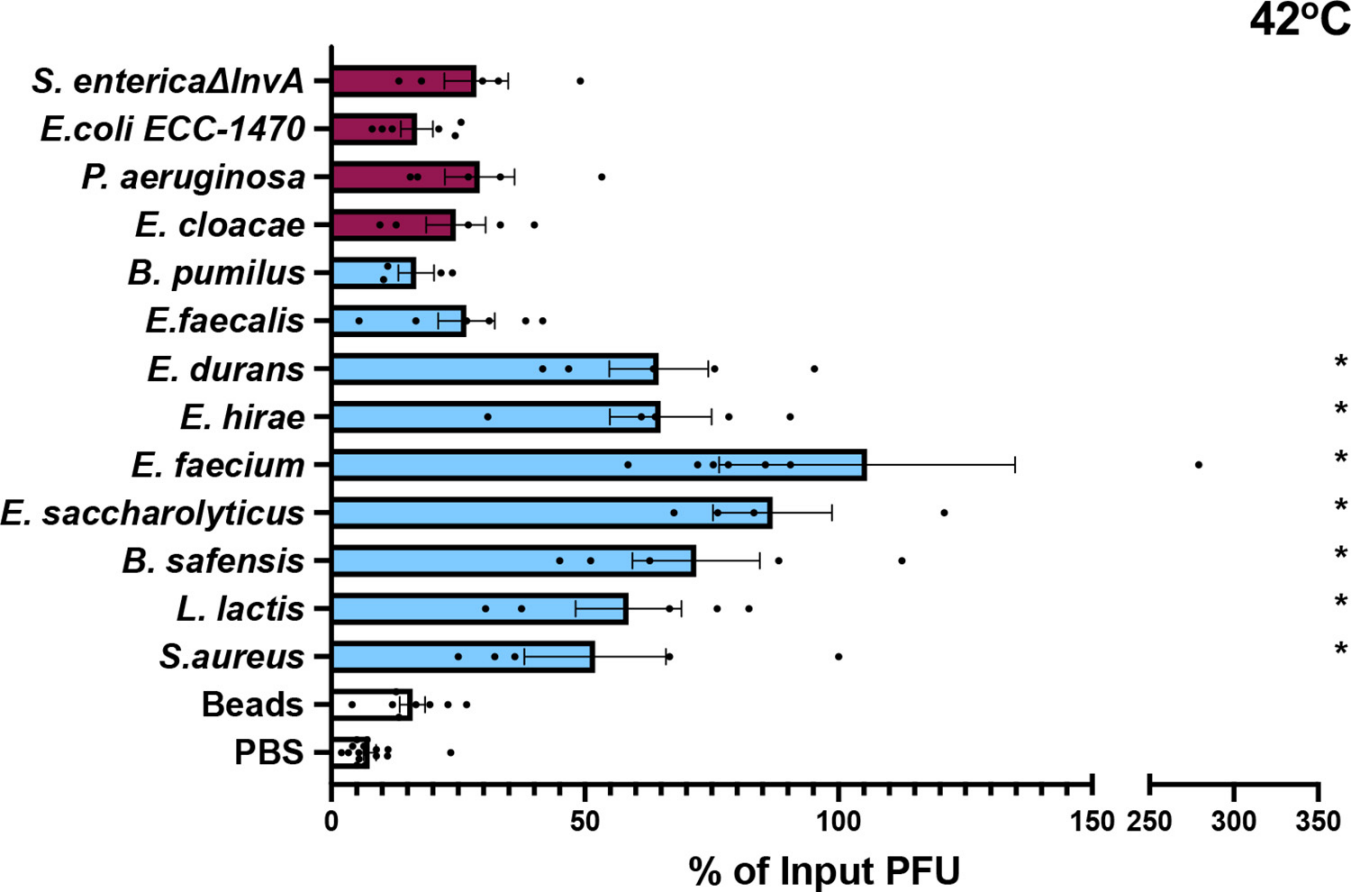
Effects of bacteria on MNV stability. Thermal stability assays were performed by incubating 1 × 10^6^ PFU MNV with PBS, streptavidin beads, or 1 × 10^9^ CFU bacteria at 42°C for 6 h. The amount of viable virus after each assay was determined by plaque assay and compared to a 4°C PBS viral titer to calculate the percentage of input PFU that remained. Data points are averages for two replicates per experiment from 4 to 16 independent experiments (*n *= 4 to 16). Bars show standard errors of the means (SEM). Statistical significance was determined by one-way ANOVA compared with the PBS samples (*, *P* < 0.05). Clear bars, controls; blue bars, Gram-positive bacteria; purple bars, Gram-negative bacteria.

**FIG 2:**
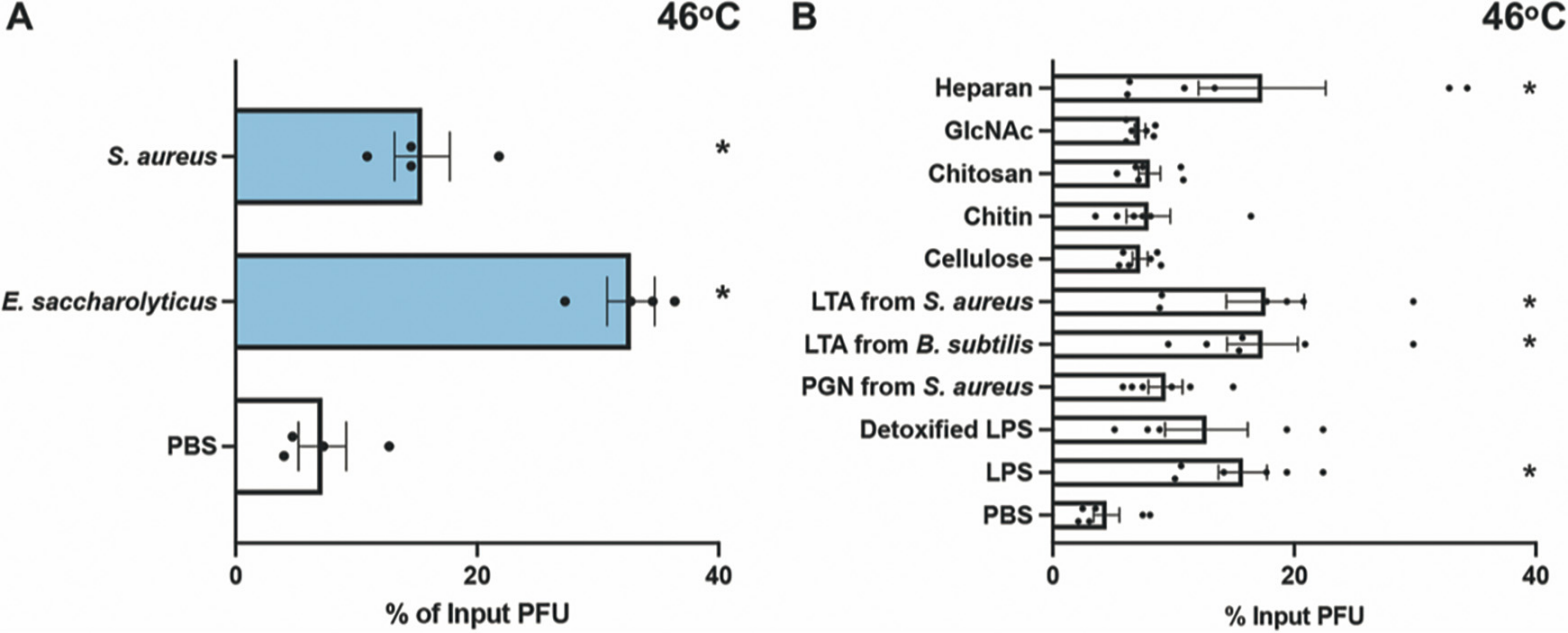
Effects of bacteria and compounds on MNV stability at high temperature. Thermal stability assays were performed by incubating 1 × 10^6^ PFU MNV with PBS, streptavidin beads, or 1 × 10^9^ CFU bacteria, or 1 mg/mL compounds at elevated temperatures. The amount of viable virus after each assay was determined by plaque assay and compared to a 4°C PBS viral titer to calculate the percentage of input PFU that remained. (A) Viral incubation with bacteria at 46°C for 4 h. Data are representative of 3 independent experiments (*n = *6). (B) Viral incubation with compounds at 46°C for 4 h. GlcNAc, *N*-acetylglucosamine; LPS, lipopolysaccharide; LTA, lipoteichoic acid; PGN, peptidoglycan. Data are representative of 3 independent experiments (*n =* 5 or 6). Bars show means and SEM. Statistical significance was determined by one-way ANOVA compared with the PBS samples (*, *P* < 0.05).

To determine if bacteria can stabilize MNV, we exposed virions to different bacterial strains and quantified viral infectivity following heat exposure. MNV and other nonenveloped RNA viral particles can be inactivated at high temperatures due to premature genome release. Previous studies have shown that bacteria and bacterial compounds are able to stabilize other nonenveloped RNA viruses at high temperatures (10–12). We first tested viral stability following incubation at 42°C for 6 h, a condition which inactivates approximately 90% of MNV infectivity (Fig. 1). The virus was mixed with phosphate-buffered saline (PBS), streptavidin beads, or bacteria and incubated at 42°C for 6 h. Prior to incubation, bacteria were washed with PBS and inactivated with UV light to prevent confounding effects of bacterial growth or contamination of subsequent plaque assays. Plaque assays were used to determine the amount of viable virus remaining compared to that in samples incubated at 4°C. The majority of Gram-positive bacterial strains increased the amount of viable virus compared with PBS or bead control (Fig. 1). However, all of the Gram-negative bacteria tested had no significant impact on viral stability compared with PBS (Fig. 1). We next examined stabilization of MNV at a higher temperature. The virus was mixed with PBS, Staphylococcus aureus, or Enterococcus saccharolyticus at 46°C for 4 h. The stabilization effect of Gram-positive bacteria was also evident at this higher temperature (Fig. 2A). The 4-h incubation at 46°C was used for all subsequent experiments.

We also determined whether bacterial surface molecules could stabilize MNV. We included surface molecules from both Gram-positive and Gram-negative bacteria as well as some nonbacterial glycans that have previously been shown to stabilize other enteric viruses (10). We incubated MNV with either PBS or 1 mg/mL of each molecule at 46°C for 4 h. We found that lipoteichoic acid (LTA), a surface molecule from Gram-positive bacteria, was able to stabilize MNV. Interestingly, we found that lipopolysaccharide (LPS), a surface molecule from Gram-negative bacteria, was also able to stabilize MNV (Fig. 2B). Overall, these data suggest that LTA may contribute to MNV stabilization by Gram-positive bacteria but that other molecules may also be sufficient for stabilization.

### MNV binds to both Gram-positive and Gram-negative bacteria.

**FIG 3:**
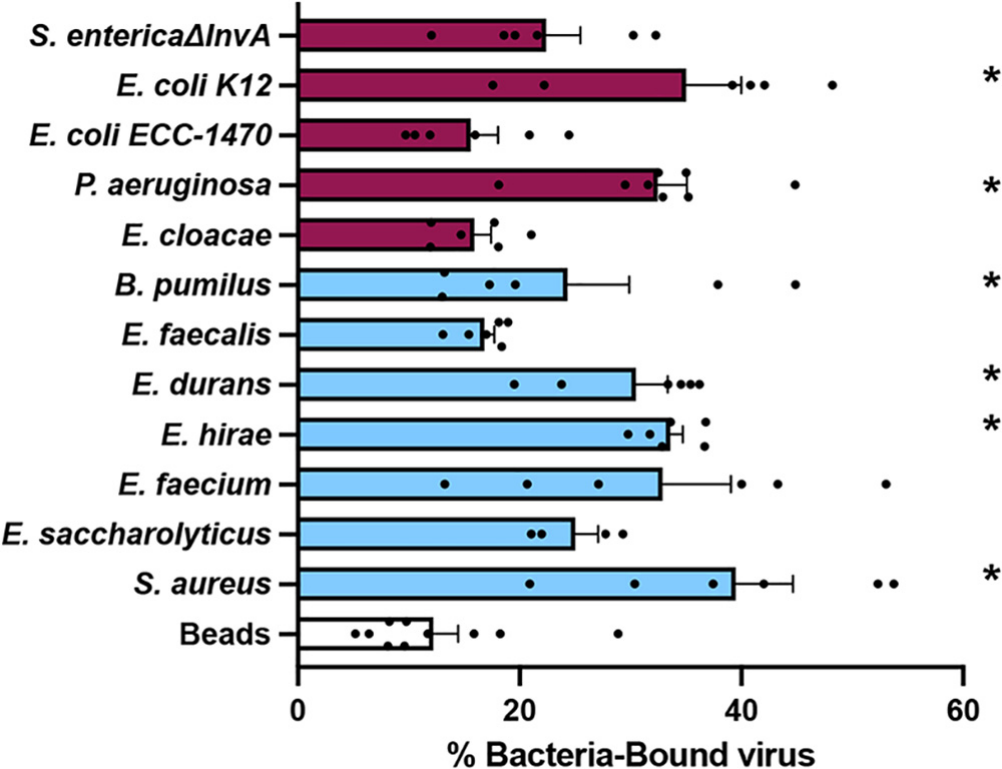
MNV binds to bacteria. ^35^S-labeled viruses were incubated with 1 × 10^9^ CFU of bacteria for 1 h at 37°C. After incubation, bacteria were spun down and washed to remove free virus. Bound virus was quantified by scintillation counting. Data are representative of 2 to 4 independent experiments (*n *= 4 to 8). Bars show means and SEM. Statistical significance was determined by one-way ANOVA compared with beads (*, *P* < 0.05). Clear bars, control; blue bars, Gram-positive bacteria; purple bars, Gram-negative bacteria.

Since bacteria were able to stabilize MNV, we determined whether MNV could interact directly with bacteria. Previously it was shown that MNV can bind to certain bacteria, although the consequences of these interactions were unclear (19). One hypothesis for the increased stabilization effects of Gram-positive bacteria is that these bacteria simply bind MNV more efficiently. We used bacterial binding assays to determine whether the increase in stabilization by Gram-positive bacteria was a result of increased binding compared to Gram-negative bacteria. To quantify binding, ^35^S-labeled MNV was incubated for 1 h with either beads or a subset of the bacteria, followed by centrifugation, washing, and scintillation counting of the bacterial pellets to determine the percentage of virus bound to the bacteria. We found that MNV binds to both Gram-positive and Gram-negative bacterial strains (Fig. 3). This indicates that not all viral binding to bacteria leads to stabilization. For example, Pseudomonas aeruginosa bound to MNV but failed to stabilize it in thermostability assays. Additionally, the increased viral stabilization by Gram-positive bacteria is not a consequence of higher binding. Overall, these results indicate that MNV can bind to both Gram-positive and Gram-negative bacteria, and although binding may be required for stabilization, it is not sufficient for stabilization.

### Incubation with bacteria does not impact MNV infectivity.

**FIG 4:**
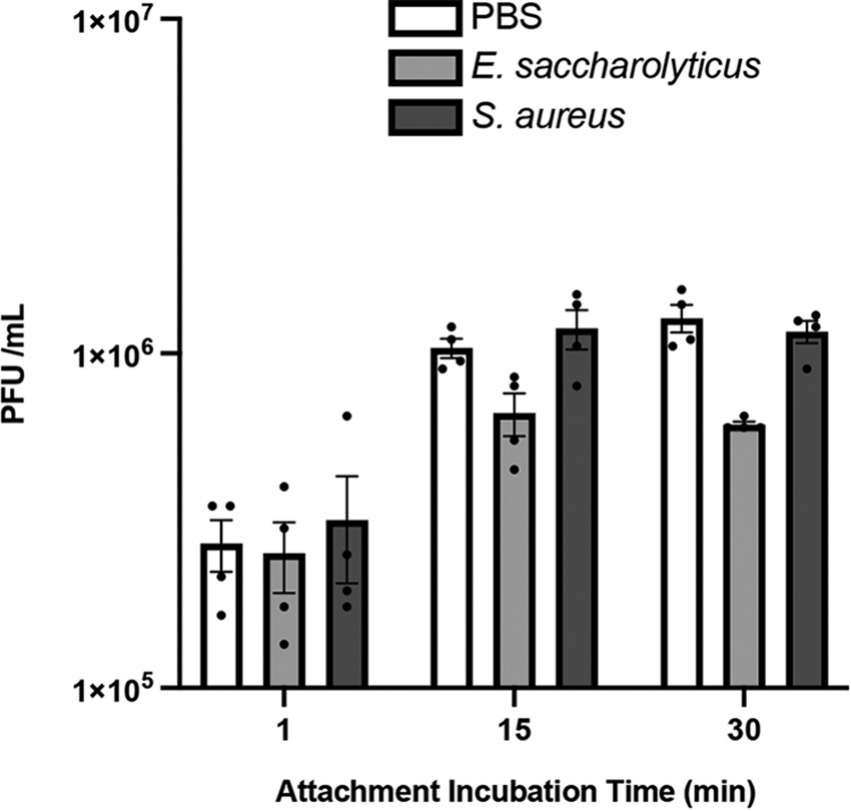
Effects of bacteria on MNV infectivity. Infectivity assays were performed by incubating 1 × 10^6^ PFU MNV with PBS or 1 × 10^9^ CFU bacteria at 37°C for 1 h prior to performing plaque assays. Each sample was incubated for 1, 15, or 30 min on the cell monolayer during the attachment period. The number of plaques was compared to that under the PBS condition for each time point. Data are representative of 2 independent experiments (*n* = 4). Bars show means and SEM. Statistical significance was determined by two-way ANOVA compared to PBS (*, *P* < 0.05).

We wanted to determine whether bacteria, in addition to stabilizing viral particles, enhance infectivity of virions in the absence of excess heat treatment. MNV was incubated with either PBS, *E. saccharolyticus*, or S. aureus at 37°C for 1 h before infecting cells for a plaque assay. For this experiment, the virus was incubated on the monolayer for 1, 5, or 15 min instead of the traditional 30-min incubation time to determine if there were any infectivity differences under more stringent conditions. We found that there were no significant differences in titer for viruses incubated with PBS or bacteria at any time point (Fig. 4). This may indicate that bacteria can prevent viral particles from becoming inactivated at high temperatures but may not alter the particles in a way that increases their ability to infect cells. Overall, these results indicate that incubation with bacteria does not make MNV more infectious for BV2 cells.

### Conditioned medium from Gram-positive bacterial cultures can stabilize MNV.

**FIG 5:**
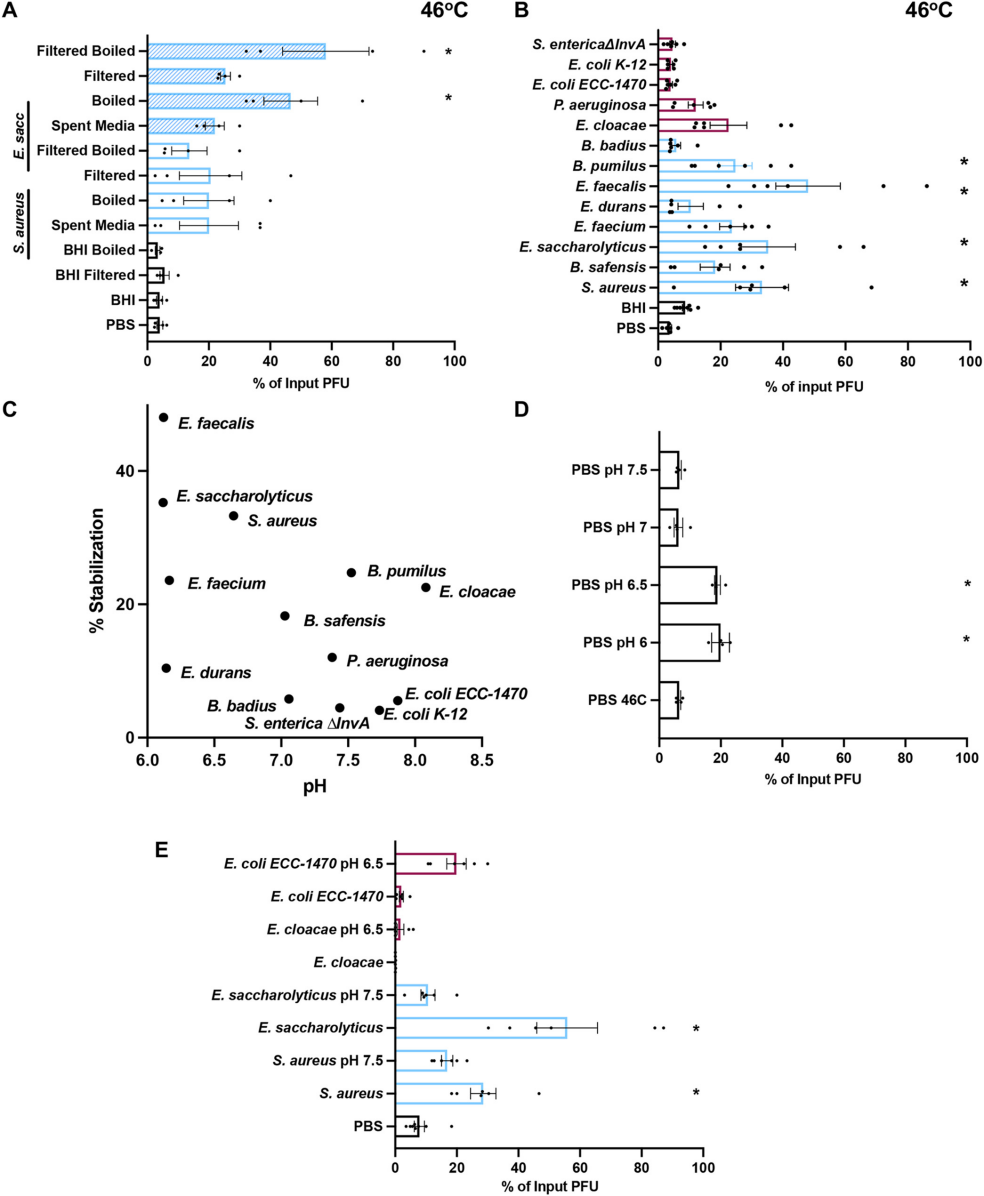
Effects of conditioned medium on MNV stability. Thermal stability assays (46°C for 4 h) were performed by incubating 1 × 10^6^ PFU MNV with PBS, BHI medium, or spent bacterial culture medium subjected to several different processing steps. (A) Thermal stability of MNV in spent medium from S. aureus and *E. saccharolyticus* either boiled for 30 min at 95°C or passed through a 0.2-μm filter. (B) Thermal stability of MNV in conditioned medium (filtered spent medium) from different bacterial strains. For both panels A and B, the amount of viable virus remaining was compared to that in a 4°C PBS control to calculate the percentage of input PFU that remained. Data are representative of 3 or 4 independent experiments (*n *= 6 to 8). Bars show means and SEM. Statistical significance was determined by one-way ANOVA compared to PBS (*, *P* < 0.05). (C) Scatterplot to test for correlation between stabilization and pH for conditioned medium from each bacterial strain. Data points are the means of stabilization values presented in panel B and the means of three independent pH measurements. Statistical significance was determined by Pearson’s correlation coefficient calculation (*R*^2^ = 0.3175, Pearson’s *r* = −0.5635, *P* = 0.0449). (D) Thermal stability of MNV in PBS with various pHs. (E) Thermal stability of MNV in conditioned medium and conditioned medium with altered pH. For panels A, B, D, and E, the amount of viable virus remaining was compared to that in a 4°C PBS control to calculate the percentage of input PFU that remained. Data are representative of 2 to 4 independent experiments (*n *= 4 to 8). Bars show means and SEM. Statistical significance was determined by one-way ANOVA compared to PBS (*, *P* < 0.05).

After observing that Gram-positive bacteria and bacterial surface molecules were able to stabilize MNV, we determined whether conditioned medium from these strains also stabilized MNV. Conditioned medium contains secreted factors or surface molecules that have sloughed off and these components could contribute to stabilization. Conditioned medium can have a variety of effects on mammalian cells and eukaryotic organisms (22–24). However, the impact of conditioned medium on viruses is unknown. First, we determined whether sterile medium, filtered medium, or boiled medium could stabilize MNV after incubation at 46°C, and we found no stabilizing activity (Fig. 5A). Next, we tested viral stability in the presence of conditioned medium for two species, *E. saccharolyticus* and S. aureus, that had significant levels of stabilization at 46°C from whole bacteria (Fig. 2A). We found that spent medium that was filtered with a 0.2-μm filter and boiled for 30 min was able to stabilize MNV, in contrast to brain heart infusion (BHI) growth medium alone, indicating that the stabilizing factor is heat stable and smaller than a whole bacterium (Fig. 5A). We then tested viral stability in the presence of conditioned medium from a larger subset of bacteria. We found that the conditioned medium from some Gram-positive bacteria were able to stabilize MNV. The conditioned medium from all of the Gram-negative bacteria tested did not have an effect on viral stability, consistent with the data from the whole-bacterium stability assay (Fig. 5B). Overall, these results indicate that the presence of whole bacteria is not required for stabilization and that the stabilizing component is heat stable.

Previous work has shown that pH can affect virion conformational changes (25, 26); therefore, we examined pH as a possible contributor to the virion stabilization phenotypes. While experiments with whole bacteria (Fig. 1) were performed after washing and resuspension in PBS at pH 7.25, it was possible that conditioned medium derived from different bacterial strains could have different pHs, which could affect MNV stability. To address potential effects of pH, we performed several experiments. First, we measured the pH of conditioned medium from each of the bacterial strains and plotted pH versus MNV stabilization values from Fig. 5B. The data indicated a minor correlation between low pH and MNV stabilization (Fig. 5C) (*R*^2^ = 0.3175, Pearson’s *r* = −0.5635, *P* = 0.0449). Second, we incubated MNV in PBS at several different pH values and performed the stability assay. We found that MNV exposed to lower pH (pH 6.0 and 6.5) had slightly higher stability than MNV exposed to standard PBS at pH 7.25 (Fig. 5D). Third, to determine whether MNV stabilization by conditioned medium could be due to pH effects, we decreased the pH of conditioned medium with stabilizing activity and increased the pH of conditioned medium without stabilizing activity, and then we quantified MNV stabilization. We chose four strains: two Gram-negative strains with conditioned medium that did not stabilize MNV (Escherichia coli ECC-1470, pH 7.8; E. cloacae, pH 8.1) and two Gram-positive strains with conditioned medium that did stabilize MNV (*E. saccharolyticus*, pH 6.1; S. aureus*;* pH 6.6). We found that lowering the pH of conditioned medium from E. coli or Enterobacter cloacae did not stabilize MNV (Fig. 5E, top), suggesting that lack of stabilizing activity is not simply due to pH effects for these Gram-negative bacteria. However, increasing the pH of conditioned medium from *E. saccharolyticus* or S. aureus reduced MNV stabilization (Fig. 5E, bottom), suggesting loss of stabilizing function with increased pH. Overall, these results suggest that MNV is not stabilized by exposure to conditioned medium from the tested Gram-negative bacteria regardless of pH condition and that MNV stabilization by conditioned medium from Gram-positive bacteria is pH dependent.

### A small, protease- and heat-stable molecule from bacterially conditioned medium is sufficient to stabilize MNV.

**FIG 6:**
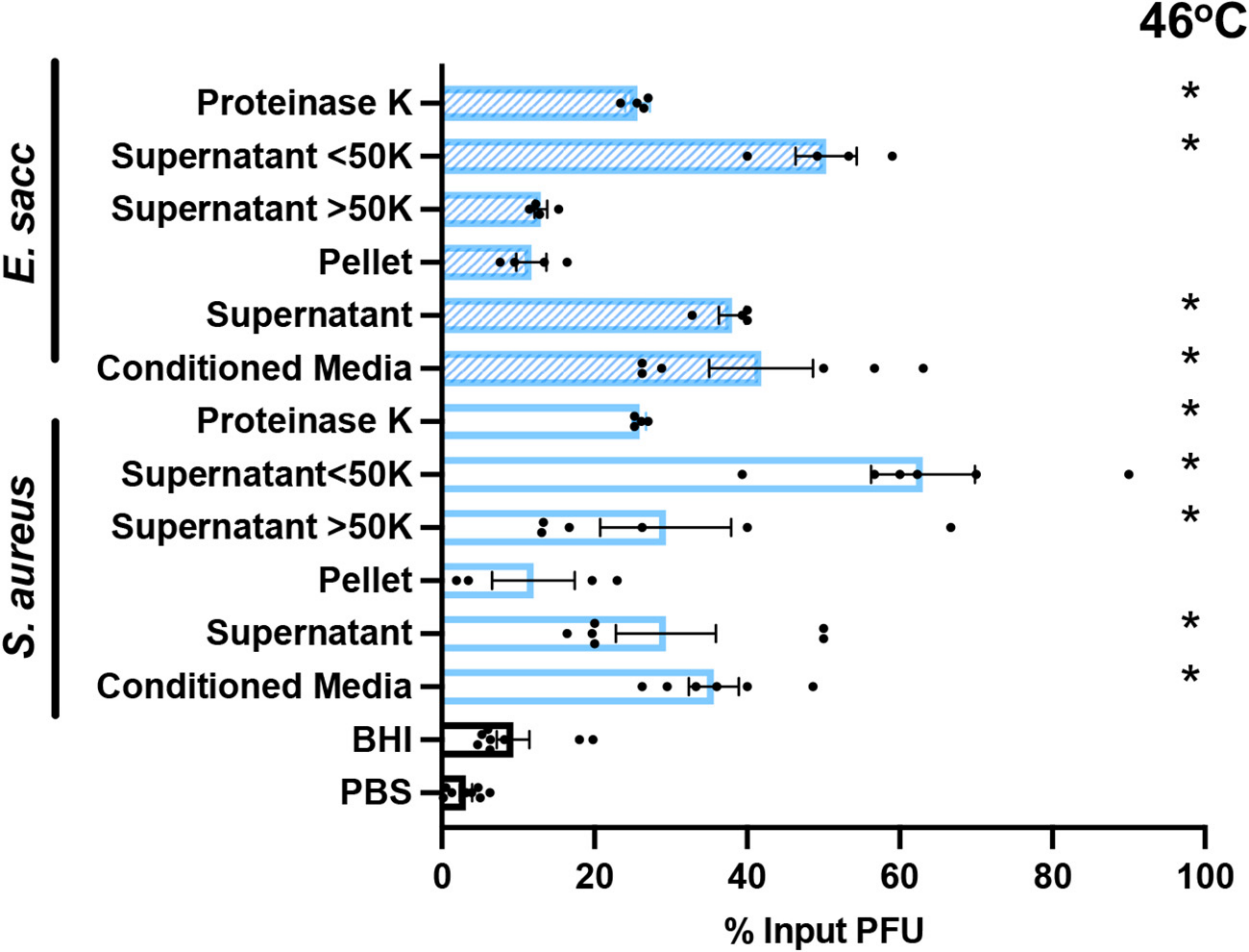
Effects of conditioned-medium treatments on stabilization of MNV. Thermal stability assays were performed by incubating 1 × 10^6^ PFU MNV with PBS, BHI, and conditioned medium from S. aureus and *E. saccharolyticus* that was filtered with a 0.2-μm filter and then separated with a 50-kDa spin column or subjected to ultracentrifugation at 150,000 × *g* for 3 h to generate supernatants and pellets. The samples were incubated with virus at 46°C for 4 h. The amount of viable virus after each assay was determined by plaque assay and compared to that in a 4°C PBS control to calculate the percentage of input PFU that remained. Data are representative of 2 to 4 independent experiments (*n *= 4 to 8). Bars show means and SEM. Statistical significance was determined by one-way ANOVA compared to PBS (*, *P* < 0.05).

Since conditioned medium from most Gram-positive bacterial strains stabilized MNV, we wanted to further define properties of the stabilizing factor. We chose the bacterial strains *E. saccharolyticus* and S. aureus from the Gram-positive group as representative bacteria. Recent evidence shows that Gram-positive bacteria can produce extracellular vesicles, lipid bilayer-enclosed particles that can contain diverse cargo such as nucleic acids, effector proteins, and enzymes (27, 28). In order to determine if extracellular vesicles or other relatively large structures impacted MNV stability, we performed ultracentrifugation and used both the pellet and supernatant in thermal stability assays. We found that, for both strains, the supernatant but not the pellet was able to stabilize MNV, indicating that the stabilizing factor may not involve extracellular vesicles or other larger structures (Fig. 6). Next, we used size exclusion spin columns to fractionate the conditioned medium. We tested both the >50-kDa and <50-kDa fractions in a thermal stability assay and found that for both strains, there was significant MNV stabilization from the <50-kDa fraction. However, for S. aureus there was also statistically significant stabilization from >50-kDa fraction (Fig. 6). This may suggest that the stabilizing factor in the conditioned medium can be a variety of sizes. Lastly, we determined whether the stabilizing factor in conditioned medium is protease sensitive. Bacterially conditioned medium was treated with proteinase K for 18 h before the thermal stability assay with MNV. We found that for both strains, the conditioned medium maintained the ability to stabilize MNV after protease treatment. This suggests that the stabilizing molecule in the conditioned medium is a heat- and protease-stable molecule that is relatively small.

## DISCUSSION

While it is established that the gut microbiota can influence MNV infection *in vivo* (16–18), there are still outstanding questions regarding the role of specific bacteria and underlying mechanisms. Previous studies have shown that proviral effects of the gut microbiota on MNV infection is lost in mice lacking certain innate immune factors. This suggests that the gut microbes may alter the immune system in a way that promotes viral infection (17). In addition to the role that bacteria may play in modulating the immune response, we are interested in the direct effects of bacteria on MNV infection. Here, we show that certain bacterial species, bacterial surface molecules, and bacterially conditioned medium can increase the stability of MNV.

Our data indicate that Gram-positive but not Gram-negative bacteria are able to stabilize MNV. We found that most Gram-positive bacterial strains were able to stabilize MNV (Fig. 1). Although the mechanism underlying this stabilization is unclear, we found that LTA isolated from Gram-positive bacteria (S. aureus and Bacillus subtilis) was able to stabilize MNV on its own (Fig. 2B). Since LTA is an important cell wall polymer in Gram-positive bacteria, these data suggest that it may play a role in stabilization (29). Curiously, while exposure to Gram-negative bacteria did not stabilize MNV (Fig. 1), exposure to LPS from Gram-negative bacteria did (Fig. 2B). We do not know the reason for the stabilizing activity of LPS, but it could be due to higher concentrations used in the purified LPS assays compared with amounts on the surfaces of bacteria or the fact that purified LPS forms micelles.

Although we found that most Gram-positive bacterial strains stabilized MNV while Gram-negative bacteria did not (Fig. 1), we found that both groups were able to bind MNV (Fig. 3). We hypothesize that binding is a minimum requirement for stabilization, as all of the strains that were able to stabilize MNV also bound to the virus. However, we also hypothesize that MNV binding is not sufficient for stabilization, as illustrated by the Gram-negative bacterial strains. Work with other enteric viruses in the picornavirus family also supports these ideas. Erickson et al. (11) and Dhalech et al. (13) showed that poliovirus or coxsackievirus B3 binding to bacteria does not always correlate with the ability of the bacteria to increase infectivity or stability. In addition to whole bacteria stabilizing MNV by a potential direct interaction mechanism, we have shown that conditioned medium is also able to stabilize MNV (Fig. 5). We found that the conditioned medium from most Gram-positive bacteria was able to stabilize MNV (Fig. 5). Further, we found that for the conditioned medium from *E. saccharolyticus* and S. aureus, the largest amount of stabilizing activity was found in the fraction of medium with molecules of <50 kDa and the stabilizing effect was maintained after protease treatment, suggesting that the relevant factor is not a protease-sensitive protein (Fig. 6). Prior studies have shown that a range of factors, including bile salts, divalent cations, and pH, can influence conformational changes in norovirus particles (25, 26, 30, 31). Further work is needed to determine the exact identity and biochemical properties of the stabilizing components of conditioned medium.

Lastly, we found that the stabilizing effects of conditioned medium on MNV did not increase infectivity in a plaque assay (Fig. 4). This may indicate that bacteria play more of a role in maintaining viability for a viral particle in a certain environment or length of time than in making the particle more infectious. Other enteric viruses, such as coxsackievirus B3 and poliovirus, exhibit a correlation between increased infectivity and stability (10, 13). This may indicate that the mechanism for MNV stabilization is distinct from that for other enteric viruses.

Overall, this study illuminates the role that specific bacterial species and bacterial surface components play in MNV infection and uncovers MNV stabilization by bacterially conditioned medium. Understanding the role of bacterial species and bacterially conditioned medium in stabilizing MNV can provide insight into how MNV establishes an infection and how MNV may spread between hosts due to increased stability in the environment.

## MATERIALS AND METHODS

### Cells and viral stocks.

BV2 cells were grown in Dulbecco’s modified Eagle medium (DMEM) with 10% fetal bovine serum, 1% HEPES, and 1% penicillin-streptomycin. MNV-1.CW3 (MNV1) (5) was generated by transfecting HEK293T cells with an infectious clone plasmid followed by two rounds of amplification in BV2 cells to generate high-titer viral stocks. Viral stocks were stored at −80°C.

To quantify virus, plaque assays were performed as previously described. Briefly, virus was diluted in phosphate-buffered saline supplemented with 100 μg/mL CaCl_2_ and 100 μg/mL MgCl_2_ (PBS+) and added to BV2 cells for 30 min at 37°C to facilitate attachment. Overlays containing 7.5% methylcellulose, MEM, 10% fetal bovine serum, 1% penicillin-streptomycin, and 1% HEPES were used and removed after 72 h.

Radiolabeled virus was generated by propagating MNV in the presence of [^35^S]cysteine-methionine. BV2 cells plated in 15-cm plates were infected at a multiplicity of infection (MOI) of 0.05 for 3 h before adding 0.36 mCi of [^35^S]cysteine-methionine (Perkin Elmer) for 45 h. The media and cells were freeze-thawed 3 times and centrifuged to remove cell debris. Supernatants were centrifuged through a 30% sucrose cushion at 27,000 rpm for 3 h at 4°C in SW28 rotor. Viral pellets were resuspended in 350 μL of 10% *N*-lauryl sarcosine and left at room temperature for 2 h. Virus was added to a CsCl solution made in PBS and adjusted to a refractive index of 1.3665. CsCl gradients were formed by ultracentrifugation at 35,000 rpm for 40 h at 12°C in an SW55 rotor. Individual fractions were collected from the top of the gradients, followed by scintillation counting and performing plaque assays to determine virus-containing fractions. The purity of virus was confirmed by SDS-PAGE and phosphorimaging. Viral fractions were dialyzed against PBS at 4°C before being stored at −80°C in glass tubes.

### Bacterial strains and conditioned medium.

Bacterial strains were obtained from ATCC or from the ceca of mice as described previously (11). Note that the E. cloacae strain used here is a non-ATCC strain from a teaching lab and has unknown H antigen status (5, 11). Overnight cultures were inoculated from glycerol stocks in BHI medium. The optical density at 600 nm (OD_600_) was determined for each culture with a spectrophotometer (Eppendorf BioPhotometer D30) to determine the CFU required for each experiment. The required volume of bacteria was pelleted, washed twice, and resuspended in PBS+. Bacteria were UV inactivated prior to use in assays by exposing the bacteria to UV light for 30 min. UV inactivation conditions were confirmed by plating on BHI agar.

Conditioned medium was generated by growing overnight cultures of each bacterial strain before different types of processing for each conditioned-medium experiment. For Fig. 5A, bacteria were pelleted by centrifugation, and the supernatant is referred to as spent medium. The spent medium was then either filtered using a 0.2-μm filter or boiled in a 95°C heat block for 30 min. For Fig. 5B, bacteria were pelleted and the supernatant was then filtered with a 0.2-μm filter. The filtered medium is referred to as conditioned medium. For Fig. 6, the conditioned medium was spun at ~150,000 × *g* for 3 h at 4°C in an ultracentrifuge. The supernatant was collected, and the pellet was resuspended in PBS+. The supernatant was then separated using a 50-kDa spin column (Millipore Sigma), and both the >50-kDa and <50-kDa fractions were collected. The conditioned medium was also treated with 0.1 mg/mL proteinase K (Invitrogen Ambion) for 18 h at 37°C (24). The enzyme was inactivated by boiling the sample for 30 min.

### Viral stability assays.

To determine whether bacteria impacted the stability of MNV, 1 × 10^6^ PFU of MNV was mixed with either PBS+, 1 × 10^9^ CFU bacteria, 1 mg/mL of compounds/molecules, or 200 μL bacterial conditioned medium and incubated at 42°C for 6 h or 46°C for 4 h. A control sample of virus in PBS+ was placed at 4°C for the duration of the experiment. After incubation, plaque assays were performed on both the heat-exposed samples and the 4°C control sample using BV2 cells to determine the amount of viable virus before and after heat treatment. The percentage of input PFU remaining after heat exposure was calculated by dividing the titer of each sample by that of the control sample.

### Binding assays.

The bacterial binding assay was performed as previously described for poliovirus. Approximately 3,500 cpm of ^35^S-radiolabeled virus was mixed with PBS or 1 × 10^9^ CFU of bacteria and incubated at 37°C for 1 h. Binding reactions were done in the presence of 0.1% bovine serum albumin (BSA) to prevent nonspecific binding. After incubation, bacteria were pelleted and washed with PBS+ to remove any unbound virus. Counts per minute for both input and bacteria were obtained by scintillation counting to determine the amount of virus that was bound to bacterial cells.

### Infectivity assays.

To determine the effect of conditioned medium on the infectivity of MNV, 1 × 10^6^ PFU was incubated with either PBS or conditioned medium for 1 h at 37°C. Tenfold dilutions of the preincubated MNV media were plated as in a standard plaque assay. The plaque assay plates were incubated for either 1 min, 5 min, or 15 min and washed, and overlay was added. The titers of the virus incubated with PBS and with the conditioned medium were compared for each time point.

### Data analysis.

Statistical analyses were performed using GraphPad Prism software. All one-way analyses of variance (ANOVA) were performed with Dunnett’s multiple-comparison *post hoc* test. For scatterplot data, the *P* value (two-tailed), *R*^2^, and Pearson’s *r* were calculated and are reported in the figures.
